# Impact of retraction force magnitudes on mobility of maxillary canines: a split-mouth design

**DOI:** 10.1186/s40510-022-00408-5

**Published:** 2022-05-02

**Authors:** Nehal F. Albelasy, Yasser L. Abdelnaby

**Affiliations:** Department of Orthodontics, Faculty of Dentistry, Mansoura University, Mansoura, 35511 Ad-Daqahliyah Egypt

**Keywords:** Canine retraction, Canine mobility, Retraction force, Periotest, PTVs

## Abstract

**Objective:**

Prospective evaluation of the maxillary canine mobility during retraction using two different force levels over 5 months of retraction.

**Materials and methods:**

Thirty patients indicated for maximum retraction of maxillary canines with age range of 14.7–18.9 years were included in the study. After complete leveling and alignment and immediately before canine retraction, the mobility of the maxillary canines was measured using the Periotest device and repeated monthly. A split-mouth design was applied where on the one side, the retraction force was 100 g, while on the other side 200 g of force. Four subgroups were investigated: A1 (R3 100 g), A2 (L3 200 g), B1 (R3 200 g) and B2 (L3 100 g). The total amount of canine retraction was measured for each side using the pre- and post-retraction dental casts.

**Results:**

The collected data were normally distributed. ANOVA test showed insignificant statistical difference in Periotest values (PTVs) among the four subgroups pre-retraction and monthly *p* > 0.05. However, each group showed a statistically significant difference in PTVs over the 5 months. The independent sample *t* test showed a statistical insignificant difference in PTVs between the 100 g and 200 g retraction force. Pearson correlation of the PTVs to the period of retraction was statistically significant *p* < 0.05 while being in significant to the retraction force *p* > 0.05.

**Conclusion:**

Increasing the retraction force of maxillary canines up to 200 g of force does not significantly increase the teeth mobility during orthodontic treatment. There is a positive correlation between the PTVs and the duration of tooth movement regardless the magnitude of force.

## Introduction

The supporting structure of the tooth “periodontium” includes the gingiva, alveolar bone, cementum and periodontal ligaments (PDLs). The PDL is a thin collagen membrane that transmits applied forces on the crown to the surrounding alveolar bone. According to Schwartz [1], upon application of the orthodontic force, the PDL is folded on the compression side and stretched on the tension side with resultant bone resorption and deposition. However, it has recently been demonstrated that the distribution of compressive and tensile strains in the periodontal tissues is more complex than initially believed [2]. This remodeling process is repeated resulting in a reduced stiffness of the PDL and increased tooth mobility with movement of the tooth along the direction of the orthodontic force [3]. It has been settled that force magnitude is one of the factors that determine the extent of the hyalinization areas.

In the past, most researchers claimed that a range of force magnitude results in a maximum rate of tooth movement, while below this range little movement occurs and above this range tooth movement is slowed down [4]. According to Quinn [5], most clinical strategies to move teeth were based on the assumption that rate of movement is sensitive to changes in force magnitude and for a given tooth there is a force that will move that tooth at a maximum rate. More recent studies have shown that no correlation was found between force magnitudes and tooth movement [6, 7].

Tooth mobility is one of the methods for assessing the biomechanical characteristics of the PDL [8]. Tooth mobility is also affected by the remodeling process of the PDL, the anatomical variations in the PDL space and alveolar bone height. The primary outcome of this split-mouth designed study was to compare the effect of using two force magnitudes on mobility of maxillary canine during retraction. The secondary outcome was to measure the resultant total amount of canine retraction done utilizing these force magnitudes.

## Materials and methods

The present study was conducted on 30 consecutive patients (22 females, 8 males) with the age range of 14.7–18.9 years and diagnosed for class II division 1 malocclusion or bimaxillary protrusion without crowding in the maxillary arch who were indicated for extraction of maxillary first premolars. The exclusion criteria were established as existence of periodontal diseases, bone resorption or inability to maintain good oral hygiene. The Research Ethics Committee of the Faculty of Dentistry, Mansoura University, Egypt approved the study protocol. All the patients were informed of the procedures and signed the informed consent.

The patients were hierarchically distributed with 1:1 allocation ratio for groups A and B. In group A, the right-side canine was retracted by 100 gm of force and the left side by 200 gm, where in group B, the right-side canine was retracted by 200 gm of force and the left side by 100 gm.

**Fig. 1:**
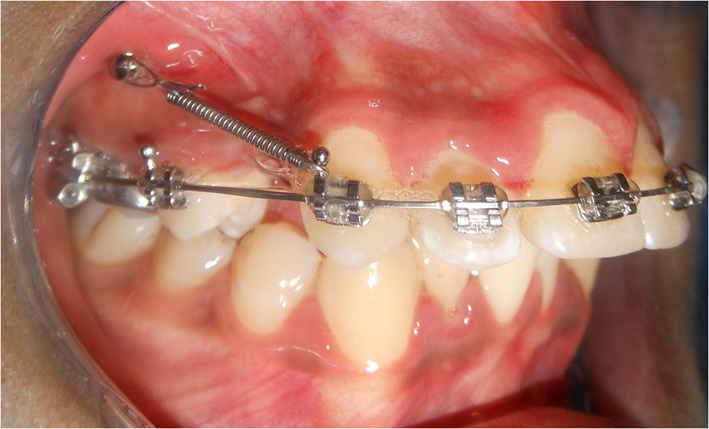
Miniscrew for maxillary canine retraction using NiTi coil spring

All patients were treated with fixed metal orthodontic appliance of Roth prescription: 0.022-inch slot size brackets. Leveling and alignment were done utilizing different sequence of arch wires following the extraction of maxillary first premolars. Orthodontic miniscrews of 1.6 mm diameter and 8 mm length (Jeil, Seoul, Korea) were self-drilled between the maxillary second premolars and the first permanent molars for maxillary canine retraction. The miniscrews were inserted at 6–8 mm from the alveolar crest and 80°–90° to the surface of the alveolar bone. A NiTi-closed coil spring was used for maxillary canine retraction on 0.017 × 0.025-inch stainless steel arch wire (Fig. 1). The retraction force was adjusted according to the allocated side.

The Periotest device was used to check the mobility of the maxillary canine immediately before retraction and repeated monthly for 5 months. The Periotest device was used according to the manufacturer’s instructions where the patients head was adjusted for making the maxillary canines perpendicular to the floor. The sleeve of the handpiece of the Periotest was perpendicularly positioned at less than 4 mm distance from the middle of the incisal–labial third of the canines. The measurements were repeated five times, and the mean was calculated for each reading. The data were divided into four subgroups according to the side and the retraction force of the maxillary canines as the following:

Group A: A1 (R3 100 g): the maxillary right canine retracted by 100 g of force.
  A2 (L3 200 g): the maxillary left canine retracted by 200 g of force.
Group B: B1 (R3 200 g): the maxillary right canine retracted by 200 g of force.
  B2 (L3 100 g): the maxillary left canine retracted by 100 g of force.

**Fig. 2:**
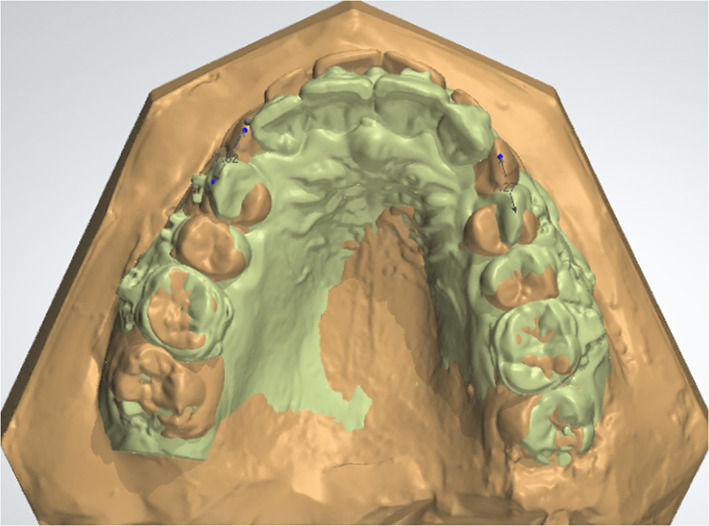
Ortho Analyzer™ software program superimposing the pre- and post-retraction dental casts

Immediately before maxillary canine retraction (T0) and after 5 months (T6), impressions were taken and poured. The casts were scanned and superimposed using Ortho Analyzer™ software program of the 3Shape Ortho System. The total amount of canine retraction was measured from the cusp tip of the maxillary canine of T0 to the same point in T6 for both sides (Fig. 2).

### Statistics

The Periotest values (PTVs) were collected, and the mean of the five measurements for every tooth was calculated. Statistical analysis was performed using the SPSS version 20.0 software for Windows (IBM, USA). All measurements were tested for normality using Shapiro–Wilk’s test. Means and standard deviations of the PTVs of maxillary canine mobility were determined before and during retraction using 100 and 200 g of force over 5-month period. *N*-way ANOVA test and least significant difference (LSD) were performed to investigate the effect of retraction force, side of canine and duration of retraction on the canine mobility represented by the PTVs. Independent sample t test was performed comparing the PTVs of maxillary canines retracted by 100 g and 200 g of force over the 5-month period. Pearson correlation was used to test if there is a correlation between the Periotest measurements and the force of retraction and the duration of retraction. Independent sample *t* test was used to compare the total amount of canine retraction measured regarding the 100 and 200 g of retraction force. Significance for all statistical tests was predetermined at *p* < 0.05.

## Results

**Fig. 3:**
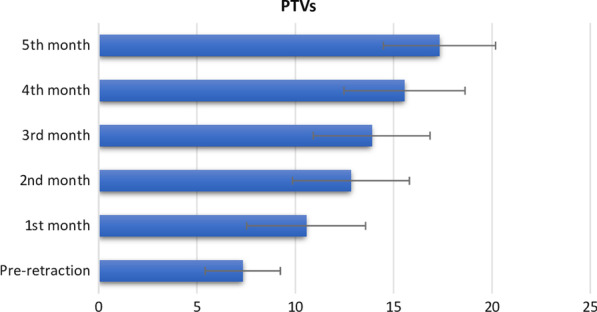
Means and standard deviation of all PTVs of the maxillary canines’ pre-retraction and monthly

Means and standard deviation of the whole PTVs of the maxillary canines’ pre-retraction and monthly are presented in Fig. 3. Means, standard deviation and the mean changes in the PTVs of the right and left canines loaded by either 100 or 200 g of force are presented in Table 1. The table shows a significant increase in the PTVs after 5 months of retraction for all groups *p* ˂ 0.05. The maxillary left canine loaded by 100 g of force showed the highest PTV 17.60 ± 2.67. The maxillary left canine loaded by 200 g of retraction force showed the highest change in the PTVs (10.40 ± 2.41). The maxillary left canine retracted by 100 gm of force showed the lowest change in the PTVs (9.50 ± 3.06). ANOVA test showed insignificant statistical difference between the four groups before retraction and in every month *p* > 0.05 (Table 2). However, ANOVA and LSD tests revealed that each group showed a statistically significant difference in PTVs over the 5 months *p* ˂ 0.05 (Table 2). The right and left sides were pooled, and an independent sample t test was performed comparing the PTVs of maxillary canine subjected to 100 and 200 g of retraction forces. The results showed a statistical insignificant difference in the PTVs *p* > 0.05 (Table 3). Pearson correlation of the PTVs with the period of retraction was statistically significant *p* ˂ 0.05, while with the amount of retraction force, it was insignificant *p* > 0.05 (Table 4). Independent sample t test showed insignificant difference in the total amount of canine retraction using either 100 or 200 g of retraction force *p* > 0.05 (Table 5).

**Table 1:** Means, standard deviations, the changes in the PTVs prior to canine retraction and the 5th month and the *p* value of the paired Student’s *t* test

	Pre-retraction Mean ± SD	5th month Mean ± SD	5th month—pre-retraction Mean ± SD	*p* value
R3 100 gm	7.60 ± 1.96	17.20 ± 3.58	9.60 ± 2.46	0.000*
L3 100 gm	7.70 ± 1.70	17.60 ± 2.67	9.50 ± 3.06	0.000*
R3 200 gm	6.90 ± 2.33	17.00 ± 3.56	10.10 ± 2.41	0.000*
L3 200 gm	7.10 ± 1.79	17.50 ± 1.51	10.40 ± 2.41	0.000*

*Statistically significant at *p* value ˂ 0.05

**Table 2:** Means and standard deviations of Periotest values of the right and left canines loaded by 100 and 200 g of force and the *p* value of ANOVA test

	R3 100 gm	L3 100 gm	R3 200 gm	L3 200 gm	*p* value
Pre-retraction	7.60 ± 1.96^abcd^	7.70 ± 1.70^abcd^	6.90 ± 2.33^abcde^	7.10 ± 1.79^abcde^	0.762
1st Month	10.50 ± 3.03^ab^	11.00 ± 2.82^acd^	9.80 ± 2.49^acde^	10.90 ± 3.90^acde^	0.820
2nd Month	12.50 ± 2.71^b^	14.70 ± 3.37^ad^	12.22 ± 3.17^be^	12.00 ± 2.00^bcde^	0.137
3rd Month	13.50 ± 3.50^ac^	14.40 ± 2.88^bd^	13.30 ± 3.49^ace^	14.30 ± 2.11^abce^	0.802
4th Month	15.20 ± 3.79^ad^	16.20 ± 2.86^c^	14.90 ± 3.87^ad^	15.90 ± 1.52^abd^	0.779
5th Month	17.20 ± 3.58^abc^	17.60 ± 2.67^abd^	17.00 ± 3.56^abce^	17.50 ± 1.51^abce^	0.967
*p* value	0.000*	0.000*	0.000*	0.000*	

Means with the same superscript letters in column are significantly different at *p* ˂ 0.05 according to the least significant test (LSD) test. *Statistically significant at *p* value ˂ 0.05

**Table 3:** Means, standard deviations, the changes in the PTVs prior to canine retraction and monthly till the 5th month regarding the force magnitude and the *p* value of the independent sample *t* test

	PTV**—**100 g Mean ± SD	PTV**—**200 g Mean ± SD	Md of PTV Mean ± SD	*p* value
Pre-retraction	7.65 ± 1.79	7.00 ± 2.02	0.65 ± 0.60	0.289
1st Month	10.75 ± 2.86	10.35 ± 3.23	0.40 ± 0.97	0.681
2nd Month	13.60 ± 3.19	12.05 ± 2.59	1.55 ± 0.92	0.099
3rd Month	13.95 ± 3.15	13.80 ± 2.86	0.15 ± 0.95	0.876
4th Month	15.70 ± 3.31	15.40 ± 2.91	0.30 ± 0.99	0.762
5th Month	17.40 ± 3.09	17.25 ± 2.67	0.15 ± 0.91	0.870

**Table 4:** Pearson correlation and *p* value of the PTVs to the duration of retraction and the force of retraction

	Duration of retraction	Force of retraction
*PTVs*		
*r* value	0.747	0.079
*p* value	0.000*	0.220

*Statistically significant at *p* value ˂ 0.05

**Table 5:** Means, standard deviations and the *p* value of the independent sample *t* test of the total amount of canine retraction by 100 and 200 g of force

	Mean ± SD 100 g	Mean ± SD 200 g	*t*	*df*	*p* value
Total amount of canine retraction	6.26 ± 0.52	6.52 ± 0.58	1.518	38	0.137

## Discussion

Orthodontic tooth movement occurs as a result of a cellular remodeling process of the periodontium in response to the applied orthodontic load. This response depends upon the intensity and duration of the applied force which in turn produces stresses and strains in the surrounding tissues. Many studies [5, 6, 9] compared different force magnitudes from 10 to 300 cN for either tipping or bodily movement of different teeth using different appliance systems. These studies have shown that there was no correlation between the magnitude of force and the rate of tooth movements.

Quinn et al. [5] advocated three major problems that complicate clinical studies of force magnitude and tooth movement including inability to maintain the type of tooth movement caused by the appliances used, nonlinear time-dependent course of tooth movement following appliance activation and the measurements errors as well as the large variations in the rate of tooth movement between patients and even quadrants in an individual patients. Pilon et al. [9] found that the individual characteristics are the major decisive factor in determining the rate of orthodontic tooth movement rather than the magnitude of force.

Since tooth mobility is considered as one of the methods for evaluating the biomechanical characteristics of the PDL, the present study aimed to investigate the effect of force magnitudes on mobility of the maxillary canines and if they were affecting the rate of retraction. Two magnitudes of force, 100 g and 200 g, were used for maxillary canine retraction with 0.017 × 0.025″ SS wire over a period of 5 months using the Periotest device. The Periotest has been approved to be a simple and accurate method for clinical evaluation and quantification of the teeth mobility and, accordingly, the viscoelastic behavior of the periodontium [10, 11]. To overcome the individual variability, a split-mouth design was applied where on the one side the canine was retracted by 100 g and on the other side of the same patient, the canine was retracted by 200 g of force. These sides were reversed in the other group. The PTVs were calculated for each canine tooth immediately before starting application of retraction forces and analyzed statistically for all groups and subgroups, and they were found to be insignificant as presented in Table 2 (*p* > 0.05).

The PTVs of the maxillary canines were recorded monthly using the Periotest device. Previous studies [10, 12] reported a range of 5–10 of the PTVs of healthy incisor teeth, while others [11] reported 10.8 for healthy upper central incisor. The PTVs of the maxillary canines in the present study ranged from 4 to 10 immediately before starting retraction. This can be explained by the absence of anterior crowding in the selected cases, thus diminishing the effect of leveling and alignment forces. It also might be due to the good periodontal support of the maxillary canines in comparison with the central incisors.

The increase in PTVs over the 5 months indicates the increase in remodeling process where bone resorption occurs as a result of the light continuous force. However, no difference was found between the retraction forces, 100 g and 200 g, which might indicate that both forces are within the physiologic limit where the process and the rate of bone remodeling were similar for both forces. This was also confirmed by the correlation found between the PTVs and the duration of retraction (*r* = 0.747) where there was no correlation found between the PTVs and the magnitude of the retraction forces (*r* = 0.079). Also, the results showed that there was no statistically significant difference in the total amount of canine retraction between the two groups with a 6.26 ± 0.52 mm total mean of retraction for the 100 g and 6.25 ± 0.58 mm for the 200 g group as presented in Table 5 (*p* > 0.05).

## Conclusion

1. Increasing the retraction force of maxillary canines up to 200 g of force does not increase the teeth mobility during orthodontic treatment
2. There is a positive correlation between the PTVs and the duration of tooth movement regardless the magnitude of force being either 100 or 200 g.
3. No significant difference in the amount of canine retraction by using either 100 g or 200 g of force.

### Limitations

1. Evaluation of the tipping degree of canine retraction regarding different force levels was not considered.
2. The available research data suggesting the ideal timing of mobility assessment regarding the remodeling process was not established yet.
3. Comparison with different force mechanics was not considered.

## Data Availability

The collected and analyzed datasets are available from the corresponding author on reasonable request.
